# Does the Combination of Abdominal Obesity and Vitamin D Deficiency Increase the Risk of Death in Individuals Aged 50 or Older? Evidence From the ELSA Study

**DOI:** 10.1111/dom.70839

**Published:** 2026-05-06

**Authors:** Joyce da Silva Milliati, Thaís Barros Pereira da Silva, Roberta de Oliveira Máximo, Mariane Marques Luiz, Sara Souza Lima, Andrew Steptoe, Cesar de Oliveira, Tiago da Silva Alexandre

**Affiliations:** ^1^ Gerontology Department Federal University of Sao Carlos Sao Carlos Brazil; ^2^ Postgraduate Programme in Gerontology Federal University of Sao Carlos Sao Carlos Brazil; ^3^ Postgraduate Programme in Physical Therapy Federal University of Sao Carlos Sao Carlos Brazil; ^4^ Department of Behavioural Science and Health University College London London UK; ^5^ Department of Epidemiology and Public Health University College London London UK

**Keywords:** 25(OH)D, abdominal obesity, ELSA study, mortality, vitamin D

## Abstract

**Aims:**

Despite the known association between abdominal obesity (AO) and vitamin D deficiency, it remains unclear whether this combination is associated with higher mortality risk. We investigated whether the coexistence of AO and vitamin D deficiency elevates the risk of death.

**Materials and Methods:**

Five thousand, five hundred twenty participants from the ELSA Study, aged ≥ 50 years, were followed for 6 years. AO was defined by waist circumference (> 102 cm for men and > 88 cm for women). Vitamin D levels, measured via serum 25‐hydroxyvitamin D [25(OH)D] concentrations, were categorised as sufficient (≥ 50 nmol/L), insufficient (30–50 nmol/L), and deficient (< 30 nmol/L). At baseline, participants were classified according to AO (non‐abdominal obesity [NAO] and AO) and 25(OH)D status (sufficiency [VDS], insufficiency [VDI], and deficiency [VDD]), creating six groups: NAO/VDS, NAO/VDI, NAO/VDD, AO/VDS, AO/VDI, and AO/VDD. Cox regression models adjusted for sociodemographic, behavioural, and clinical characteristics estimated mortality risk.

**Results:**

The NAO/VDI and NAO/VDD groups were associated with a 91% (HR = 1.91; 95% CI: 1.37–2.66) and 81% higher risk of death (HR = 1.81; 95% CI: 1.25–2.62), respectively. The AO/VDS and AO/VDI groups had a 47% (HR = 1.47; 95% CI: 1.02–2.14) and 50% higher mortality risk (HR = 1.50; 95% CI: 1.01–2.23), respectively, compared to the NAO/VDS group. The highest mortality risk was observed in the AO/VDD group (HR = 2.23; 95% CI: 1.50–3.33).

**Conclusions:**

AO combined with 25(OH)D deficiency increases mortality risk in older adults. Early identification and management of these conditions could reduce this risk.

## 1 Introduction

Abdominal obesity (AO) is characterised by the accumulation of visceral fat in the abdomen region and is identified by a waist circumference (WC) exceeding 102 cm for men and 88 cm for women [1]. Sex differences in abdominal adipose tissue distribution are evident across adulthood. Men show a predominance of abdominal adiposity that increases with age, whereas women exhibit mainly gluteofemoral adiposity, with ageing associated with a progressive shift towards abdominal fat accumulation [2, 3]. WC is an accessible measure of abdominal fat [2], even in the face of these sex differences, and is linked to adverse outcomes such as dyslipidaemia [4], cardiovascular disease [4], and mortality [5, 6].

Besides AO, serum vitamin D concentrations tend to decline with age, with different patterns between sexes, earlier in women, around age 50, and later in men, around age 70 [7, 8]. A reduced bioavailability of the vitamin D precursor (7‐dehydrocholesterol) occurs due to decreased skin thickness and a drop in vitamin D receptor (VDR) numbers in cells during ageing [7, 9]. This leads to a diminished capacity to synthesise and distribute vitamin D in tissues [10], thereby increasing the risk of vitamin D deficiency, characterised by serum 25‐hydroxyvitamin D [25(OH)D] < 30 nmol/[7, 11], affecting approximately 15% of older people [12].

Studies have demonstrated an inverse relationship between increasing abdominal fat and decreasing serum 25(OH)D levels [2, 13, 14]. VDR expression in adipocytes may be one mechanism that mediates this connection [2]. Obese individuals exhibit reduced expression of enzymes involved in vitamin D metabolism. To compensate for this imbalance, VDRs sequester circulating 25(OH)D in adipose tissue, creating a vitamin D reservoir that reduces its bioavailability and potentially leads to vitamin D deficiency [2, 15].

The coexistence of AO and vitamin D deficiency can have systemic consequences and increase the risk of death. Excess abdominal fat is associated with chronic low‐grade inflammation, insulin resistance, and oxidative stress [3, 16]. Moreover, low levels of 25(OH)D impair immune regulation and can heighten the inflammatory response, particularly in individuals with obesity [17]. Together, these processes worsen metabolic dysfunction and systemic inflammation and disrupt cellular homeostasis, which may raise the risk of death [15, 17, 18].

Although some studies have indicated a link between obesity, vitamin D deficiency, and a higher risk of death, evidence supporting this connection remains limited in older adults. Saliba et al. monitored 174 781 Israelis aged 20 and over for 4 years and found that individuals with obesity (body mass index [BMI] ≥ 30 kg/m^2^) had a higher risk of death when their 25(OH)D levels were below 40 nmol/[15]. Furthermore, the authors observed an increased risk of death among individuals of normal weight (BMI < 25 kg/m^2^) when 25(OH)D levels were < 61 nmol/L, compared with those with sufficient levels (≥ 75 nmol/L). Song et al. tracked 40 058 Americans aged 20 and over for 12 years and reported a higher risk of death among those with obesity (BMI ≥ 30 kg/m^2^) and AO (WC > 102 cm for men and > 88 cm for women) who also had vitamin D insufficiency (> 30 to ≤ 50 nmol/L) or deficiency (≤ 30 nmol/L), compared with individuals with levels above 50 nmol/L [18].

Evidence about whether the combined conditions of AO and 25(OH)D deficiency may raise the risk of death in older adults remains limited. Therefore, we investigated whether the coexistence of AO and vitamin D deficiency increases the risk of death among individuals aged 50 or older.

## 2 Materials and Methods

### 2.1 Study Population

The English Longitudinal Study of Ageing (ELSA) is a prospective panel study with a representative sample of community‐dwelling English individuals aged 50 years or older. The ELSA Study started in 2002, with participants who previously took part in the Health Survey for England in 1998, 1999, and 2001 [19]. In the ELSA Study, home interviews are conducted every 2 years using questionnaires, and additional visits by the nursing team are carried out every 4 years to collect biomarkers, as well as to perform physical examinations and functional performance tests. Details of the study design have been described in a previous publication [20].

**FIGURE 1:**
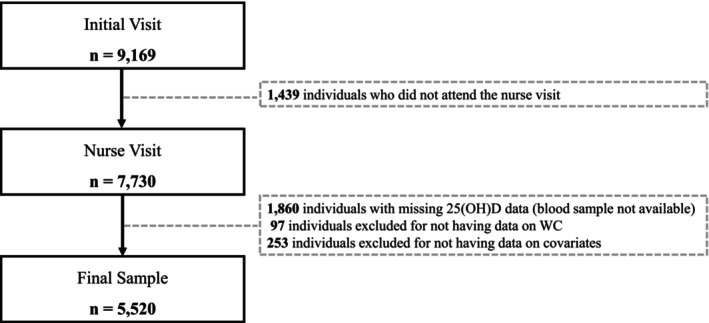
Flowchart of participant selection at baseline in the English longitudinal study of ageing.

The baseline for this study corresponds to Wave 6 of the ELSA Study (2012/2013), when serum 25(OH)D concentrations were first collected. Wave 6 of the ELSA Study (2012/2013) included 9169 individuals. Of these, 1439 did not attend the nurse visit. Blood collection was not performed in participants with clotting or bleeding disorders, a history of seizures, or current use of anticoagulant medication [21], resulting in 1860 individuals with missing serum 25(OH)D data. Among the remaining 5870 individuals, 97 were excluded for missing WC data, and another 253 were excluded due to missing covariate information. Consequently, the final sample included 5520 individuals (Figure 1).

### 2.2 Mortality

**FIGURE 2:**
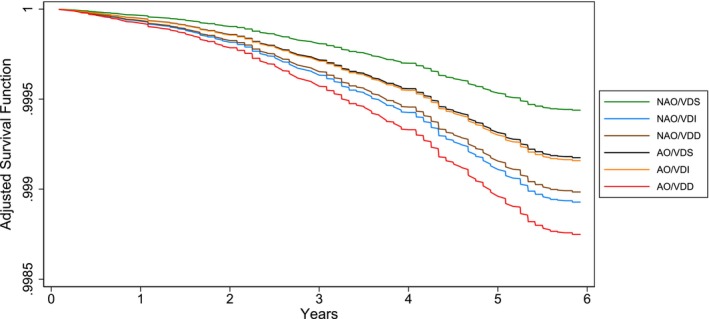
Survival curve based on abdominal obesity status and serum 25(OH)D levels, derived from the final Cox proportional hazards model. *Note:* NAO/VDS: Non‐abdominal obesity/vitamin D sufficiency; NAO/VDI: Non‐abdominal obesity/vitamin D insufficiency; NAO/VDD: Non‐abdominal obesity/vitamin D deficiency; AO/VDS: Abdominal obesity/vitamin D sufficiency; AO/VDI: Abdominal obesity/vitamin D insufficiency; AO/VDD: Abdominal obesity/vitamin D deficiency.

Death dates for participants who died during the specified period were obtained from the UK National Health Service Mortality Registry. Mortality data were collected in Waves 7 (2014/2015), 8 (2016/2017), and 9 (2018/2019), covering a six‐year period (Figure 2).

### 2.3 AO

AO was assessed by measuring WC. In the ELSA Study, this measurement was performed using a flexible, non‐stretchable tape measure placed at the midpoint between the last rib and the upper margin of the iliac crest, and it was recorded twice. If a discrepancy greater than three centimetres was found between the two measurements, a third measurement was taken. For analysis, the average of the two valid measurements, or if necessary, the two closest measurements was used. AO was defined as WC > 102 cm for men and > 88 cm for women [1].

### 2.4 Serum Concentrations of Vitamin D

Serum 25(OH)D concentrations at baseline were measured in blood samples collected by the nursing team and analysed at the Royal Victoria Infirmary in the United Kingdom using the DiaSorin Liaison immunoassay, which has an analytical sensitivity of 7.5 nmol/L and a coefficient of variation of 8.7%–9.4%. All assays were performed in duplicate, and the laboratory participated in the Vitamin D External Quality Assessment Scheme [7]. Serum concentrations were classified according to the Institute of Medicine criteria: sufficiency (> 50 nmol/L), insufficiency (> 30 to ≤ 50 nmol/L), and deficiency (≤ 30 nmol/L) [11].

### 2.5 Classification of Groups

At baseline, participants were divided into six groups based on the presence of AO and serum 25(OH)D levels: non‐abdominal obesity/vitamin D sufficiency (NAO/VDS); non‐abdominal obesity/vitamin D insufficiency (NAO/VDI); non‐abdominal obesity/vitamin D deficiency (NAO/VDD); abdominal obesity/vitamin D sufficiency (AO/VDS); abdominal obesity/vitamin D insufficiency (AO/VDI); and abdominal obesity/vitamin D deficiency (AO/VDD).

### 2.6 Covariates

Covariates were selected based on a theoretical framework derived from previous studies documenting the determinants of AO and vitamin D deficiency, as well as their associations with each other and with mortality [15, 18].

The sociodemographic variables considered were age (in years), sex, ethnicity (white or non‐white), marital status (with conjugal life [married individuals or those in a stable union] or without conjugal life [separated, divorced, or widowed individuals]), family wealth, and schooling. Wealth was divided into quintiles using the British standard, based on the household's total wealth (including savings, investments, property, and other assets). Schooling was classified according to the British educational system (> 13 years, 12–13 years, ≤ 11 years) [20].

The behavioural characteristics of interest included smoking status (non‐smoker, ex‐smoker, or smoker), frequency of alcohol consumption (rarely/never, frequently, daily, or did not respond), and physical activity level. Physical activity was assessed using the Physical Activity and Sedentary Behaviour Assessment Questionnaire (PASBAQ), administered as part of the Health Survey for England [22]. Participants reported the frequency (once a week, more than once a week, one to three times a month, or hardly ever or never) and intensity (vigorous, moderate, or light) of physical activity. Physical activity levels were classified as follows: vigorous (any vigorous activity at least once a week), moderate (moderate activity at least once a week), low (only light activity at least once a week), and inactive (no weekly activity) [23].

Clinical conditions were documented based on self‐reported diagnoses of hypertension, diabetes mellitus, cancer, heart disease, lung disease, stroke, osteoporosis, and osteoarthritis.

Depressive symptoms were defined as a score of ≥ 4 on the eight‐item Centre for Epidemiologic Studies Depression Scale (CES‐D) [24, 25]. Memory was assessed using immediate and delayed recall tests, in which participants were asked to recall a word list immediately after exposure and again after a five‐minute delay. Performance was measured by the total number of words correctly recalled, ranging from 0 to 20, with higher scores indicating better cognitive performance [25].

The biochemical markers of interest were total cholesterol, LDL, HDL, and triglycerides levels (mg/dL), treated as continuous variables [26].

Grip strength was measured in kilograms to assess muscle strength and treated as a continuous variable [27]. BMI was calculated as weight in kilograms divided by height in square metres (kg/m^2^) and was treated as a continuous variable [28].

The season in which blood samples were collected to measure serum 25(OH)D concentration was recorded and classified as spring (March to May), summer (June to August), autumn (September to November), or winter (December to February) [2, 7]. Vitamin D supplementation was also included as a control variable.

### 2.7 Ethical Aspects

The ELSA Study received approval from the National Research Ethics Service (London Multicentre Research Ethics Committee—MREC 01/2/91), and all participants provided informed consent.

### 2.8 Statistical Analyses

Descriptive statistics were used to assess participants' baseline characteristics. Quantitative variables were reported as means and standard deviations, and qualitative variables as proportions. Differences based on AO status and serum 25(OH)D levels were analysed using the chi‐square test and ANOVA with Tukey's post hoc test. In addition, a comparative analysis was conducted between participants included in the study and those excluded because of missing baseline data. All deaths occurring during the six‐year follow‐up period were considered.

The Kaplan–Meier method was used to plot survival curves and assess associations between AO status and serum 25(OH)D concentrations. Differences between the curves were evaluated using the log‐rank test. Cox regression models were used to examine whether the coexistence of AO and vitamin D deficiency is a risk factor for mortality. The interaction between AO and serum 25(OH)D status and sex was formally tested using the Wald test, and no statistically significant interaction was observed (*p* = 0.24).

Initially, a univariate analysis was conducted, guided by a theoretical framework. Variables with *p* ≤ 0.20 in this analysis were included in the multivariable model. In the final model, associations with *p* < 0.05 were considered statistically significant. In the Cox regression model, the NAO/VDS category served as the reference category. All analyses were performed using Stata 17 statistical software (StataCorp, College Station, TX, USA).

## 3 Results

Among the 5520 participants at baseline, 24.9% were in the non‐abdominal obesity/vitamin D sufficiency (NAO/VDS) group, 14.8% in the non‐abdominal obesity/vitamin D insufficiency (NAO/VDI) group, 9.9% in the non‐abdominal obesity/vitamin D deficiency (NAO/VDD) group, 18.9% in the abdominal obesity/vitamin D sufficiency (AO/VDS) group, 17.0% in the abdominal obesity/vitamin D insufficiency (AO/VDI) group, and 14.5% in the abdominal obesity/vitamin D deficiency (AO/VDD) group. A total of 419 deaths (7.6%) occurred during the six‐year follow‐up, with 17.4% in the NAO/VDS group, 18.6% in the NAO/VDI group, 14.1% in the NAO/VDD group, 15.5% in the AO/VDS group, 14.3% in the AO/VDI group, and 20.1% in the AO/VDD group.

Baseline sample characteristics are presented in Tables 1 and 2. The mean age of participants was 66 years. The sample was predominantly female (55.2%), white (97.3%), in a conjugal relationship (67.0%), with lower educational attainment (38.0%), and with higher wealth (22.9%). Regarding lifestyle habits, participants were mainly ex‐smokers (50.2%), frequently consumed alcohol (39.9%), and had moderate levels of physical activity (47.9%) (Table 1). The most common clinical conditions were overweight (42.2%), osteoarthritis (38.9%), and hypertension (37.4%) (Table 2).

**TABLE 1:** Sociodemographic and behavioural characteristics of 5520 participants at baseline according to abdominal obesity status and serum concentrations of 25(OH)D, ELSA Study (2012/13).

	Total (*n* = 5520)	NAO/VDS (*n* = 1375)	NAO/VDI (*n* = 819)	NAO/VDD (*n* = 549)	AO/VDS (*n* = 1042)	AO/VDI (*n* = 936)	AO/VDD (*n* = 799)
Age, (years)	66.6 ± 8.9	66.0 ± 8.5	65.9 ± 9.2	66.2 ± 9.9	67.1 ± 8.2^a^, ^b^	67.3 ± 8.6^a^, ^b^	67.3 ± 9.4^a^, ^b^
Age, (%)							
50–59 years	23.6	24.4	27.1	31.5^a^	19.6^a^, ^b^, ^c^	19.4^a^, ^b^, ^c^	23.3^c^
60–69 years	41.2	43.6	39.1	34.8^a^	42.9^c^	43.8^c^	38.4
70–79 years	26.5	25.3	25.9	21.5	30.0^c^	27.1	27.0
≥ 80 years	8.7	6.7	7.9	12.2^a^	7.5^c^	9.7	11.3^a^
Sex, (%)							
Female	55.2	49.5	46.2	51.4	59.9^a^, ^b^, ^c^	60.3^a^, ^b^, ^c^	65.3^a^, ^b^, ^c^
Ethnicity, (%)							
Non‐white	2.7	1.0	3.1^a^	6.6^a^, ^b^	1.3^c^	2.1^c^	4.9^a^, ^d^, ^e^
Marital status, (%)							
Without conjugal life	33.0	27.0	33.7^a^	41.6^a^, ^b^	27.6^b^, ^c^	34.4^a^, ^d^	42.2^a^, ^b^, ^d^, ^e^
Schooling, (%)							
> 13 years	33.1	40.4	41.6	33.5^a^, ^b^	26.4^a^, ^b^, ^c^	27.7^a^, ^b^	26.9^a^, ^b^
12–13 years	28.8	28.0	28.7	27.0	30.2	29.6	28.9
≤ 11 years	38.0	31.6	29.7	39.5^a^, ^b^	43.4^a^, ^b^	42.7^a^, ^b^	44.2^a^, ^b^
Wealth, (%)							
Highest quintile	22.9	31.0	26.6	19.1^a^, ^b^	22.0^a^	19.1^a^, ^b^	13.5^a^, ^b^, ^d^, ^e^
2nd quintile	18.3	14.5	15.9	23.1^a^, ^b^	16.9^c^	21.2^b^	22.7^a^, ^b^, ^d^
3rd quintile	20.8	21.0	18.9	18.0	23.0	21.1	21.0
4th quintile	22.1	23.9	26.8	17.7^a^, ^b^	24.4^c^	20.2^a^, ^b^	16.7^a^, ^b^, ^d^
Lowest quintile	13.9	7.9	9.5	19.9^a^, ^b^	12.1^a^, ^c^	16.1^a^, ^b^	24.4^a^, ^b^, ^d^, ^e^
Not applicable	2.0	1.7	2.3	2.2	1.6	2.3	1.7
Smoking, (%)							
Non‐smoker	38.5	40.4	41.8	35.7	37.7	38.5	34.8^b^
Ex‐smoker	50.2	50.8	45.2	40.6^a^	55.8^b^, ^c^	53.1^b^, ^c^	50.3^c^
Smoker	11.3	8.8	13.0^a^	23.7^a^, ^b^	6.5^b^, ^c^	8.4^b^, ^c^	14.9^a^, ^b^, ^d^, ^e^
Alcohol intake, (%)							
Rarely/never	18.7	12.7	14.8	22.4^a^, ^b^	17.5^a^	22.4^a^, ^b^	27.9^a^, ^b^, ^d^
Frequently	39.9	39.9	41.3	35.5	43.0^c^	40.5	36.5^d^
Daily	33.5	42.2	35.9^a^	29.2^a^	34.2^a^	28.2^a^, ^b^, ^d^	24.7^a^, ^b^, ^d^
Not applicable	7.9	5.2	8.0	12.9^a^, ^b^	5.3^c^	8.9^a^, ^d^	10.9^a^, ^d^
Physical activity, (%)							
High	33.3	49.8	39.2^a^	29.5^a^, ^b^	30.0^a^, ^b^	25.1^a^, ^b^	15.8^a^, ^b^, ^c^, ^d^, ^e^
Moderate	47.9	41.2	48.8^a^	49.9^a^	50.1^a^	52.5^a^	49.1^a^
Low	13.4	5.9	8.3	14.2^a^, ^b^	14.9^a^, ^b^	16.5^a^, ^b^	25.0^a^, ^b^, ^c^, ^d^, ^e^
Inactive	5.4	3.1	3.7	6.4^a^	5.0	5.9^a^	10.1^a^, ^b^, ^d^, ^e^

*Note:* Quantitative variables expressed as mean ± standard deviation (SD). Qualitative variables expressed as percentages (%).

Abbrevations: AO/VDD, Abdominal obesity/vitamin D deficiency; AO/VDI, Abdominal obesity/vitamin D insufficiency; AO/VDS, Abdominal obesity/vitamin D sufficiency; NAO/VDD, Non‐abdominal obesity/vitamin D deficiency; NAO/VDI, Non‐abdominal obesity/vitamin D insufficiency; NAO/VDS: Non‐abdominal obesity/vitamin D sufficiency.

^a^ Significantly different from non‐abdominal obesity/vitamin D sufficiency (*p <* 0.05).

^b^ Significantly different from non‐abdominal obesity/vitamin D insufficiency (*p <* 0.05).

^c^ Significantly different from non‐abdominal obesity/vitamin D deficiency (*p <* 0.05).

^d^ Significantly different from abdominal obesity/vitamin D sufficiency (*p* < 0.05).

^e^ Significantly different from abdominal obesity/vitamin D insufficiency (*p <* 0.05).

**TABLE 2:** Baseline clinical conditions, biochemical markers, anthropometric measurements, and performance measures for 5520 participants at baseline according to abdominal obesity status and serum concentrations of 25(OH)D, ELSA Study (2012/13).

	Total	NAO/VDS	NAO/VDI	NAO/VDD	AO/VDS	AO/VDI	AO/VDD
	(*n* = 5520)	(*n* = 1375)	(*n* = 819)	(*n* = 549)	(*n* = 1042)	(*n* = 936)	(*n* = 799)
Clinical conditions, (%)							
Hypertension	37.4	26.7	26.5	28.8	45.7^a^, ^b^, ^c^	47.3^a^, ^b^, ^c^	50.2^a^, ^b^, ^c^
Diabetes mellitus	9.5	5.1	4.4	4.2	12.3^a^, ^b^, ^c^	13.3^a^, ^b^, ^c^	17.9^a^, ^b^, ^c^, ^d^
Cancer	5.0	5.9	4.0	3.1	5.1	4.4	6.1
Heart disease	15.9	14.7	13.4	13.7	16.1	17.4	20.0^a^, ^b^, ^c^
Lung disease	13.9	11.2	11.5	14.4	14.7	14.4	19.0^a^, ^b^
Stroke	3.4	2.0	2.6	4.9^a^	3.4	3.9	5.5^a^, ^b^
Osteoporosis	8.0	9.8	6.6	5.8^a^	10.1^c^	6.9	6.6
Osteoarthritis	38.9	31.9	29.2	32.2	45.7^a^, ^b^, ^c^	44.7^a^, ^b^, ^c^	50.2^a^, ^b^, ^c^
Depressive symptoms	11.3	7.6	9.4	15.5^a^, ^b^	8.8^c^	12.6^a^	18.4^a^, ^b^, ^d^, ^e^
Biochemical markers, (mg/dL)							
Total cholesterol	214.2 ± 45.0	215.4 ± 42.1	218.8 ± 45.4	218.6 ± 43.6	208.4 ± 45.0^a^, ^b^, ^c^	214.5 ± 47.4^d^	211.7 ± 46.6^b^
HDL	64.3 ± 18.6	70.3 ± 19.1	68.1 ± 19.7	65.9 ± 19.1^a^	61.7 ± 17.0^a^, ^b^, ^c^	59.2 ± 16.0^a^, ^b^, ^c^, ^d^	58.5 ± 16.7^a^, ^b^, ^c^, ^d^
LDL	124.3 ± 40.1	124.2 ± 36.8	127.7 ± 41.0	127.2 ± 39.1	120.3 ± 39.9^b^, ^c^	125.8 ± 41.8^d^	122.2 ± 42.8
Triglycerides	128.9 ± 63.0	104.7 ± 47.2	115.2 ± 56.2^a^	127.8 ± 67.0^a^, ^b^	133.1 ± 59.7^a^, ^b^	148.6 ± 67.6^a^, ^b^, ^c^, ^d^	156.5 ± 69.5^a^, ^b^, ^c^, ^d^
Anthropometrics and performance measures							
Body mass index, (kg/m^2^)	28.0 ± 5.0	24.6 ± 2.8	24.9 ± 2.7	24.5 ± 2.8^b^	30.6 ± 4.0^a^, ^b^, ^c^	31.3 ± 4.3^a^, ^b^, ^c^, ^d^	32.2 ± 5.2^a^, ^b^, ^c^, ^d^, ^e^
Normal weight	27.5	52.3	49.2	53.4	4.7^a^, ^b^, ^c^	3.0^a^, ^b^, ^c^	2.9^a^, ^b^, ^c^
Underweight	0.9	1.9	0.9	2.9^b^	0.0	0.0	0.0
Overweight	42.2	43.8	47.1	41.3	45.0	39.3^b^	34.8^a^, ^b^, ^d^
Obesity	29.4	2.0	2.8	2.4	50.3^a^, ^b^, ^c^	57.7^a^, ^b^, ^c^, ^d^	62.3^a^, ^b^, ^c^, ^d^
Grip strength, (kg)	30.8 ± 11.6	32.1 ± 11.5	32.4 ± 11.3	30.7 ± 11.4	30.4 ± 11.7^a^, ^b^	30.3 ± 11.4^a^, ^b^	28.4 ± 11.9^a^, ^b^, ^c^, ^d^, ^e^
Memory performance, points	11.1 ± 3.4	11.5 ± 3.3	11.3 ± 3.4	10.7 ± 3.8^a^, ^b^	11.0 ± 3.3^a^	11.0 ± 3.2^a^	10.8 ± 3.5^a^, ^b^
Season of blood collection, (%)							
Spring	7.5	17.7	31.0	45.5^a^, ^b^	16.9^b^, ^c^	22.1^c^	40.8^a^, ^d^, ^e^
Summer	23.8	5.2	7.8^a^	13.8^a^, ^b^	4.5^b^, ^c^	7.2^b^, ^c^, ^d^	11.1^a^, ^d^, ^e^
Autum	42.3	30.1	16.8	10.6^a^, ^b^	34.0^c^	27.1^c^	12.3^a^, ^b^, ^d^, ^e^
Winter	26.4	47.0	44.4^a^	30.1^a^, ^b^	44.6^b^, ^c^	43.6^b^, ^c^, ^d^	35.8^a^, ^b^, ^d^, ^e^
Vitamin D supplementation, (%)	4.4	6.8	1.7^a^	1.6^a^	8.2^b^, ^c^	2.6^a^, ^d^	2.0^a^, ^d^

*Note:* Quantitative variables expressed as mean ± standard deviation (SD). Qualitative variables expressed as percentages (%).

Abbreviations: AO/VDD, Abdominal obesity/vitamin D deficiency; AO/VDI, Abdominal obesity/vitamin D insufficiency; AO/VDS, Abdominal obesity/vitamin D sufficiency; HDL, high‐density lipoprotein; LDL: low‐density lipoprotein; NAO/VDD, Non‐abdominal obesity/vitamin D deficiency; NAO/VDI, Non‐abdominal obesity/vitamin D insufficiency; NAO/VDS, Non‐abdominal obesity/vitamin D sufficiency.

^a^ Significantly different from non‐abdominal obesity/vitamin D sufficiency (*p <* 0.05).

^b^ Significantly different from non‐abdominal obesity/vitamin D insufficiency (*p* < 0.05).

^c^ Significantly different from non‐abdominal obesity/vitamin D deficiency (*p* < 0.05).

^d^ Significantly different from abdominal obesity/vitamin D sufficiency (*p* < 0.05).

^e^ Significantly different from abdominal obesity/vitamin D insufficiency (*p <* 0.05).

Participants in the AO/VDD group were older and more likely to be women, non‐white, not have a conjugal life, have a lower level of schooling and income, were more likely to smoke, consumed less alcohol, and were more physically inactive (Table 1). They also had a higher prevalence of hypertension, diabetes, heart disease, lung disease, stroke, osteoarthritis, and depressive symptoms, as well as lower HDL cholesterol levels, higher triglycerides levels, and a higher prevalence of obesity, along with lower average grip strength and poorer memory performance compared to those in the NAO/VDS group (Table 2).

Compared to the NAO/VDI group, participants in the AO/VDD group were older, more likely to be women, not have a conjugal life, have a lower level of schooling and income, be non‐smokers, consume less alcohol, and be more physically inactive (Table 1). They also had a higher prevalence of hypertension, diabetes, heart disease, lung disease, stroke, osteoarthritis, and depressive symptoms, as well as lower total cholesterol and HDL cholesterol levels, higher triglycerides levels, and obesity, along with lower mean grip strength and poorer memory performance (Table 2).

Compared to the NAO/VDD group, the participants in the AO/VDD group were more likely to be women and ex‐smokers (Table 1). They also had a higher prevalence of hypertension, diabetes, heart disease, and osteoarthritis, as well as lower HDL cholesterol levels, higher triglyceride levels, and obesity, and exhibited lower mean grip strength (Table 2).

Compared to the AO/VDS group, participants in the AO/VDD group were more likely to be non‐white, less likely to have a conjugal life, had a lower income, smoked more, consumed less alcohol, and were more physically inactive (Table 1). They also had a higher prevalence of diabetes, depressive symptoms, as well as lower HDL cholesterol levels, higher triglycerides levels, obesity, and had lower mean grip strength (Table 2).

Participants in the AO/VDD group were more likely to be non‐white, not have a conjugal life, have a lower income, smoke more, and be more physically inactive (Table 1). They also experienced a greater frequency of depressive symptoms and had lower mean grip strength compared to those in the AO/VDI group (Table 2).

Tables S1 and S2 present a comparative analysis of baseline characteristics between participants included and those excluded because of missing data. Excluded individuals were older, more likely to be non‐white, unmarried, and to have lower schooling and income. They were also more likely to be smokers, to drink alcohol less frequently, and to have lower levels of physical activity (Table S1). Clinically, excluded participants had a higher prevalence of hypertension, diabetes mellitus, cancer, heart disease, stroke, osteoporosis, and depressive symptoms. Biochemically, they had lower HDL and LDL cholesterol levels, higher triglycerides, and were more often obese. Functionally and cognitively, they exhibited lower grip strength and poorer memory performance (Table S2).

The final Cox regression models are presented in Table 3. Among participants without AO, those in the NAO/VDI and NAO/VDD groups had a 91% (HR = 1.91; 95% CI: 1.37–2.66) and 81% higher risk of death (HR = 1.81; 95% CI: 1.25–2.62), respectively, compared to the NAO/VDS group. Among participants with AO, the AO/VDS and AO/VDI groups had a 47% (HR = 1.47; 95% CI: 1.02–2.14) and 50% higher risk of death (HR = 1.50; 95% CI: 1.01–2.23), respectively. Notably, the AO/VDD group showed the highest risk, with a 123% higher risk of death (HR = 2.23; 95% CI: 1.50–3.33) compared to the NAO/VDS group.

**TABLE 3:** Final Cox regression model for mortality risk over 6 years based on abdominal obesity status and serum 25(OH)D levels in 5520 individuals aged 50 or older in the ELSA Study (2012–2018).

	HR	95% CI
Non‐abdominal obesity/vitamin D sufficiency (NAO/VDS)	1.00	
Non‐abdominal obesity/vitamin D insufficiency (NAO/VDI)	1.91	(1.37–2.66)
Non‐abdominal obesity/vitamin D deficiency (NAO/VDD)	1.81	(1.25–2.62)
Abdominal obesity/vitamin D sufficiency (AO/VDS)	1.47	(1.02–2.14)
Abdominal obesity/vitamin D insufficiency (AO/VDI)	1.50	(1.01–2.23)
Abdominal obesity/vitamin D deficiency (AO/VDD)	2.23	(1.50–3.33)

*Note:* Model adjusted for sex, age, schooling level, wealth, marital status, ethnicity, smoking, alcohol intake, physical activity level, hypertension, diabetes mellitus, cancer, heart disease, lung disease, stroke, osteoporosis, cholesterol levels, body mass index, depressive symptoms, vitamin D supplementation, season of blood collection, and grip strength. Variables significantly associated with increased mortality risk: age, wealth, smoking, physical activity level, diabetes mellitus, cancer, lung disease, osteoporosis, body mass index, depressive symptoms. Variables significantly associated with a protective effect for mortality (*p* < 0.05): sex, body mass index, grip strength.

Abbreviations: CI, confidence interval; HR, hazard ratio.

## 4 Discussion

Based on this large sample of English individuals, our results demonstrated that the AO/VDD group had a higher risk of death during the six‐year follow‐up period compared to the NAO/VDS group.

Previous research on younger adults has shown that obesity and vitamin D deficiency are associated with higher mortality risk. For example, Saliba et al. followed 174 781 Israelis aged ≥ 20 years for 4 years and found that obese individuals (BMI ≥ 30 kg/m^2^) had higher mortality risk only when 25(OH)D concentrations were below 40 nmol/L. For normal‐weight individuals (BMI < 25 kg/m^2^), the threshold for elevated risk was slightly higher, and overweight individuals (BMI 25–29.9 kg/m^2^) had increased risk when serum 25(OH)D concentrations were below 61 nmol/L and 48 nmol/L, respectively [15]. Similarly, Song et al., analysing 40 058 adults aged ≥ 20 years from the NHANES cohort over 12 years, reported a 53% higher risk of death among obese participants (BMI ≥ 30 kg/m^2^) with 25(OH)D deficiency (≤ 30 nmol/L), and a 57% higher risk among individuals with AO (> 102 cm for men, > 88 cm for women) and 25(OH)D deficiency, compared to non‐abdominally obese participants with sufficient 25(OH)D. Notably, normal‐weight and non‐abdominally obese participants with low 25(OH)D also had elevated mortality risk (51%–55%), similar to that of obese participants [18].

Although evidence exists in younger adults, studies in older populations remain scarce. Furthermore, differences in study design make direct comparisons difficult and limit the identification of high‐risk subgroups. Our study addresses these gaps by examining the joint impact of AO and 25(OH)D deficiency on mortality in adults aged ≥ 50 years.

One possible mechanism underlying our results is that vitamin D plays a crucial role in regulating the immune response, exerting anti‐inflammatory effects by inhibiting pro‐inflammatory cytokine production and enhancing regulatory T lymphocyte activity [29]. This regulation is impaired when serum 25(OH)D levels are deficient, thereby promoting a chronic low‐grade inflammatory state even in the absence of excess body fat [29]. Furthermore, 25(OH)D deficiency impairs endothelial function, disrupts the renin‐angiotensin system balance, and alters cellular energy metabolism, thereby increasing susceptibility to cardiovascular and metabolic disorders [17]. These effects are even more pronounced in older populations, as they are associated with muscle mass loss, reduced mobility, and a weakened immune response [7, 30]. Therefore, 25(OH)D deficiency can impair multiple physiological systems even in individuals with healthy body composition, fostering the development of chronic diseases and contributing to higher mortality rates [17, 29].

Both conditions are damaging in their own right, particularly in people with AO. However, the impact may be intensified because VDRs sequester circulating 25(OH)D when excess body fat is present, thereby reducing the vitamin's overall bioavailability and limiting its immunomodulatory effects [2, 31].

Another potential mechanism that could explain our findings is the development of an exacerbated inflammatory environment and metabolic dysfunction caused by the coexistence of AO and 25(OH)D deficiency, which may increase the risk of death. The accumulation of visceral fat is closely linked to chronic low‐grade inflammation, characterised by the continuous production of pro‐inflammatory cytokines, including interleukin‐6, tumour necrosis factor‐α, and C‐reactive protein [16]. This inflammatory environment promotes insulin resistance, oxidative stress, and mitochondrial dysfunction, undermining cellular signalling and the regulation of energy metabolism, thereby increasing the risk of mortality [3, 16].

The strengths of this study include the use of a representative sample of community‐dwelling English adults aged 50 years or older and the examination of a wide range of sociodemographic, behavioural, and clinical variables, which together enabled a more robust and consistent fit of the Cox proportional hazards models. The relatively long follow‐up period further enhanced the robustness of the findings. Our study appears to be the first to identify the combination of AO and 25(OH)D deficiency as a risk factor for death in a sample of older individuals.

The present study has limitations that should be acknowledged. First, because the ELSA sample comprised only community‐dwelling individuals, the results cannot be generalised to institutionalised populations. Another limitation is the absence of tests for determining body composition, such as dual‐energy X‐ray absorptiometry (DXA) and computed tomography, which provide greater accuracy in measuring abdominal fat [32, 33]. Although these methods are more precise, their use in population studies is impractical due to high costs and the need for specialised equipment and trained technicians. Therefore, we chose to use WC, a simple, low‐cost measure widely used to predict adverse outcomes [34]. Additionally, participants excluded from the analyses differed in certain characteristics from those included. However, the fact that AO/VDD was identified as a risk factor even in a sample with a lower prevalence of mortality predictors suggests that our results are robust. Consequently, the risk associated with AO/VDD may be underestimated, reinforcing the clinical significance of our findings. Lastly, the ELSA study does not include the collection of key biochemical markers for our research, such as serum parathyroid hormone (PTH) levels, which are inversely associated with serum 25(OH)D concentrations. Elevated PTH levels indicate secondary hyperparathyroidism, a condition that can also contribute to the accumulation of abdominal fat.

The coexistence of AO and vitamin D deficiency increases mortality risk. These findings highlight the potential benefits of routine screening and appropriate clinical management for both conditions to reduce mortality among individuals aged 50 and older.

## Author Contributions

Design: Joyce da Silva Milliati, Thaís Barros Pereira da Silva, Tiago da Silva Alexandre. Conduct/data collection: Tiago Alexandre, Cesar de Oliveira, Andrew Steptoe. Analysis: Thaís Barros Pereira da Silva, Roberta de Oliveira Máximo, Tiago Alexandre. Writing manuscript: Joyce da Silva Milliati, Thaís Barros Pereira da Silva, Roberta de Oliveira Máximo, Mariane Marques Luiz, Sara Souza Lima, Cesar de Oliveira, Tiago Alexandre.

## Funding

The ELSA Study was funded by the National Institute on Aging, part of the US National Institutes of Health (NIH), under Grant Number R01AG017644, and by UK government agencies coordinated by the National Institute for Health Research (NIHR) through the Policy Research Programme (HEI) 98_1074_03. The opinions expressed here are solely those of the authors and do not necessarily reflect the institutional views of the NIH, NIHR, or the Department of Health and Social Care. The present study was supported by the Economic and Social Research Council (Grant Number: ES/T008822/11), the National Council for Scientific and Technological Development (CNPq) (Grant No. 305338/2023‐4, awarded to TSA), and the São Paulo Research Foundation (FAPESP) (Grant No. 2024/01918‐6, also awarded to TSA). The funders had no influence on the study design, data collection and analysis, or the writing of the manuscript.

## Conflicts of Interest

The authors declare no conflicts of interest.

## Supporting information


**Table S1:** Sociodemographic and behavioural characteristics of participants included versus excluded at baseline in the ELSA Study (2012/13).
**Table S2:** Clinical conditions, biochemical markers, anthropometric measurements, and performance measures of participants included versus excluded at baseline in the ELSA Study (2012/13).

## Data Availability

Data from the ELSA Study are available from the UK Data Service to researchers who meet the criteria for access to confidential data, under the terms of the End User Licence (http://ukdataservice.ac.uk/media/455131/cd137‐enduserlicence.pdf). The data can be accessed at: http://discover.ukdataservice.ac.uk/series/?sn=200011. Contact the UK Data Service regarding access to the English Longitudinal Study of Ageing through the website (http://ukdataservice.ac.uk/help/get‐in‐touch.aspx), by phone on +44 (0)1206 872 143, or by email at help@ukdataservice.ac.uk.

## References

[1] Adults (US) NOEIEP on the I Evaluation, and Treatment of Obesity in , Clinical Guidelines on the Identification, Evaluation, and Treatment of Overweight and Obesity in Adults (National Heart, Lung, and Blood Institute, 1998).

[2] T. B. P. da Silva , M. M. Luiz , M. L. B. Delinocente , A. Steptoe , C. de Oliveira , and T. d. S. Alexandre , “Is Abdominal Obesity a Risk Factor for the Incidence of Vitamin D Insufficiency and Deficiency in Older Adults? Evidence From the ELSA Study,” Nutrients 14, no. 19 (2022): 4164, 10.3390/nu14194164. 36235815 PMC9572900

[3] P. C. Ramírez , D. C. de Oliveira , M. R. de Oliveira , et al., “Is Dynapenic Abdominal Obesity a Risk Factor for Cardiovascular Mortality? A Competing Risk Analysis,” Age and Ageing 52, no. 1 (2023): afac301, 10.1093/ageing/afac301. 36626317 PMC9831270

[4] B. Nussbaumerova and H. Rosolova , “Obesity and Dyslipidemia,” Current Atherosclerosis Reports 25, no. 12 (2023): 947–955, 10.1007/s11883-023-01167-2. 37979064

[5] A. T. da Silva , S. Scholes , J. L. Ferreira Santos , Y. A. de Oliveira Duarte , and C. de Oliveira , “Dynapenic Abdominal Obesity Increases Mortality Risk Among English and Brazilian Older Adults: A 10‐Year Follow‐Up of the ELSA and SABE Studies,” Journal of Nutrition, Health & Aging 22, no. 1 (2018): 138–144, 10.1007/s12603-017-0966-4. PMC12880444 29300433

[6] Y. H. Kim , S. M. Kim , K. D. Han , et al., “Waist Circumference and All‐Cause Mortality Independent of Body Mass Index in Korean Population From the National Health Insurance Health Checkup 2009–2015,” Journal of Clinical Medicine 8, no. 1 (2019): 72, 10.3390/jcm8010072. 30634601 PMC6352259

[7] M. Marques Luiz , M. R. de Oliveira , A. F. de Souza , et al., “Is Serum 25‐Hydroxyvitamin D Deficiency a Risk Factor for the Incidence of Slow Gait Speed in Older Individuals? Evidence From the English Longitudinal Study of Ageing,” Diabetes, Obesity and Metabolism 27 (2025): 3104–3112, 10.1111/dom.16317. PMC12046445 40083058

[8] D. Maggio , A. Cherubini , F. Lauretani , et al., “25(OH)D Serum Levels Decline With Age Earlier in Women Than in Men and Less Efficiently Prevent Compensatory Hyperparathyroidism in Older Adults,” Journals of Gerontology. Series A, Biological Sciences and Medical Sciences 60, no. 11 (2005): 1414–1419, 10.1093/gerona/60.11.1414. 16339327

[9] N. Cochar‐Soares , T. B. P. da Silva , M. M. Luiz , et al., “Vitamin D Deficiency as a Risk Factor for Cognitive Decline in Individuals Aged 50 or Older,” GeroScience 48 (2025): 2491–2503, 10.1007/s11357-025-01800-9. 40690159 PMC12972389

[10] A. Giustina , R. Bouillon , B. Dawson‐Hughes , et al., “Vitamin D in the Older Population: A Consensus Statement,” Endocrine 79, no. 1 (2023): 31–44, 10.1007/s12020-022-03208-3. 36287374 PMC9607753

[11] A. C. Ross , J. E. Manson , S. A. Abrams , et al., “The 2011 Report on Dietary Reference Intakes for Calcium and Vitamin D From the Institute of Medicine: What Clinicians Need to Know,” Journal of Clinical Endocrinology and Metabolism 96, no. 1 (2011): 53–58, 10.1210/jc.2010-2704. 21118827 PMC3046611

[12] A. Cui , T. Zhang , P. Xiao , Z. Fan , H. Wang , and Y. Zhuang , “Global and Regional Prevalence of Vitamin D Deficiency in Population‐Based Studies From 2000 to 2022: A Pooled Analysis of 7.9 Million Participants,” Frontiers in Nutrition 10 (2023): 1070808, 10.3389/fnut.2023.1070808. 37006940 PMC10064807

[13] M. Pereira‐Santos , P. R. F. Costa , A. M. O. Assis , C. a. S. T. Santos , and D. B. Santos , “Obesity and Vitamin D Deficiency: A Systematic Review and Meta‐Analysis,” Obesity Reviews: An Official Journal of the International Association for the Study of Obesity 16, no. 4 (2015): 341–349, 10.1111/obr.12239. 25688659

[14] M. Zhang , P. Li , Y. Zhu , et al., “Higher Visceral Fat Area Increases the Risk of Vitamin D Insufficiency and Deficiency in Chinese Adults,” Nutrition and Metabolism 12 (2015): 50, 10.1186/s12986-015-0046-x. 26612998 PMC4660664

[15] W. Saliba , O. Barnett‐Griness , and G. Rennert , “Obesity and Association of Serum 25(OH)D Levels With All‐Cause Mortality,” Calcified Tissue International 95, no. 3 (2014): 222–228, 10.1007/s00223-014-9885-0. 24958474

[16] P. C. Ramírez , M. R. de Oliveira , D. Capra de Oliveira , et al., “Dynapenic Abdominal Obesity as a Risk Factor for Metabolic Syndrome in Individual 50 Years of Age or Older: English Longitudinal Study of Ageing,” Journal of Nutrition, Health & Aging 27, no. 12 (2023): 1188–1195, 10.1007/s12603-023-2039-1. PMC12880461 38151869

[17] J. P. Sutherland , A. Zhou , and E. Hyppönen , “Vitamin D Deficiency Increases Mortality Risk in the UK Biobank : A Nonlinear Mendelian Randomization Study,” Annals of Internal Medicine 175, no. 11 (2022): 1552–1559, 10.7326/M21-3324. 36279545

[18] S. Song , Y. Yuan , X. Wu , et al., “Additive Effects of Obesity and Vitamin D Insufficiency on All‐Cause and Cause‐Specific Mortality,” Frontiers in Nutrition 9 (2022): 999489, 10.3389/fnut.2022.999489. 36337642 PMC9634746

[19] J. Mindell , J. P. Biddulph , V. Hirani , et al., “Cohort Profile: The Health Survey for England,” International Journal of Epidemiology 41, no. 6 (2012): 1585–1593, 10.1093/ije/dyr199. 22253315

[20] A. Steptoe , E. Breeze , J. Banks , and J. Nazroo , “Cohort Profile: The English Longitudinal Study of Ageing,” International Journal of Epidemiology 42, no. 6 (2013): 1640–1648, 10.1093/ije/dys168. 23143611 PMC3900867

[21] M. M. Luiz , R. Máximo , D. C. Oliveira , et al., “Association of Serum 25‐Hydroxyvitamin D Deficiency With Risk of Incidence of Disability in Basic Activities of Daily Living in Adults >50 Years of Age,” Journal of Nutrition 150, no. 11 (2020): 2977–2984, 10.1093/jn/nxaa258. 32937653 PMC7675030

[22] D. H. T. de Carvalho , S. Scholes , J. L. F. Santos , C. de Oliveira , and T. d. S. Alexandre , “Does Abdominal Obesity Accelerate Muscle Strength Decline in Older Adults? Evidence From the English Longitudinal Study of Ageing,” Journals of Gerontology. Series A, Biological Sciences and Medical Sciences 74, no. 7 (2019): 1105–1111, 10.1093/gerona/gly178. 30107482 PMC6580692

[23] S. Scholes , N. Coombs , Z. Pedisic , et al., “Age‐ and Sex‐Specific Criterion Validity of the Health Survey for England Physical Activity and Sedentary Behavior Assessment Questionnaire as Compared With Accelerometry,” American Journal of Epidemiology 179, no. 12 (2014): 1493–1502, 10.1093/aje/kwu087. 24863551 PMC4051878

[24] D. E. Steffick , Documentation of Affective Functioning Measures in the Health and Retirement Study (Institute for Social Research, University of Michigan, 2000).

[25] M. B. Ofstedal and G. Fisher , Documentation of Cognitive Functioning Measures in the Health and Retirement Study (Institute for Social Research, University of Michigan, 2005).

[26] D. O. Fedder , C. E. Koro , and G. J. L'Italien , “New National Cholesterol Education Program III Guidelines for Primary Prevention Lipid‐Lowering Drug Therapy,” Circulation 105, no. 2 (2002): 152–156, 10.1161/hc0202.101971. 11790693

[27] D. E. Alley , M. D. Shardell , K. W. Peters , et al., “Grip Strength Cutpoints for the Identification of Clinically Relevant Weakness,” Journals of Gerontology. Series A, Biological Sciences and Medical Sciences 69, no. 5 (2014): 559–566, 10.1093/gerona/glu011. 24737558 PMC3991145

[28] Obesity: Preventing and Managing the Global Epidemic. Report of a WHO Consultation,” World Health Organization Technical Report Series 894, no. 1–12 (2000): 1–253. 11234459

[29] C. Aranow , “Vitamin D and the Immune System,” Journal of Investigative Medicine 59, no. 6 (2011): 881–886, 10.2310/JIM.0b013e31821b8755. 21527855 PMC3166406

[30] M. L. B. Delinocente , M. M. Luiz , D. C. de Oliveira , et al., “Are Serum 25‐Hydroxyvitamin D Deficiency and Insufficiency Risk Factors for the Incidence of Dynapenia?,” Calcified Tissue International 111, no. 6 (2022): 571–579, 10.1007/s00223-022-01021-8. 36109388 PMC9613743

[31] W. Saliba , O. Barnett , H. S. Rennert , and G. Rennert , “The Risk of All‐Cause Mortality Is Inversely Related to Serum 25(OH)D Levels,” Journal of Clinical Endocrinology and Metabolism 97, no. 8 (2012): 2792–2798, 10.1210/jc.2012-1747. 22648653

[32] O. E. Osayande , G. N. Azekhumen , and E. O. Obuzor , “A Comparative Study of Different Body Fat Measuring Instruments,” Nigerian Journal of Physiological Sciences 33, no. 2 (2018): 125–128. 30837764

[33] E. Völgyi , F. A. Tylavsky , A. Lyytikäinen , H. Suominen , M. Alén , and S. Cheng , “Assessing Body Composition With DXA and Bioimpedance: Effects of Obesity, Physical Activity, and Age,” Obesity (Silver Spring) 16, no. 3 (2008): 700–705, 10.1038/oby.2007.94. 18239555

[34] I. Janssen , P. T. Katzmarzyk , and R. Ross , “Waist Circumference and Not Body Mass Index Explains Obesity‐Related Health Risk,” American Journal of Clinical Nutrition 79, no. 3 (2004): 379–384, 10.1093/ajcn/79.3.379. 14985210

