# Supplementary material for: Does the Combination of Abdominal Obesity and Vitamin D Deficiency Increase the Risk of Death in Individuals Aged 50 or Older? Evidence From the ELSA Study
Source: Diabetes Obes Metab. 2026 May 6;28(8):6607–17. doi: 10.1111/dom.70839 (PMC13341344; doi:10.1111/dom.70839)
Supplement: Supplementary file 1 — Table S1: Sociodemographic and behavioural characteristics of participants included versus excluded at baseline in the ELSA Study (2012/13). Table S2: Clinical conditions, biochemical markers, anthropometric measurements, and performance measures of participants included versus excluded at baseline in the ELSA Study (2012/13). [file DOM-28-6607-s001.docx]

**Supplementary Material**

**Does the combination of abdominal obesity and vitamin D deficiency increase the risk of death in individuals aged 50 or older? Evidence from the ELSA Study**

**Supplementary Table 1.** Sociodemographic and behavioural characteristics of participants included versus excluded at baseline in the ELSA Study (2012/13).

**Supplementary Table 2.** Clinical conditions, biochemical markers, anthropometric measurements, and performance measures of participants included versus excluded at baseline in the ELSA Study (2012/13).

**Supplementary Table 1.** Sociodemographic and behavioural characteristics of participants included versus excluded at baseline, ELSA Study (2012/13).

|  | **Included**  **(n = 5,520)** | **Excluded**  **(n = 3,649)** |
| --- | --- | --- |
| **Age, (years)** | 66.6 ± 8.9 | 69.5 ± 10.6† |
| **Age, (%)** |  |  |
| 50 – 59 years | 23.6 | 20.8† |
| 60 – 69 years | 41.2 | 32.3† |
| 70 – 79 years | 26.5 | 26.9 |
| ≥ 80 years | 8.7 | 20.0† |
| **Sex, (%)** |  |  |
| Female | 55.2 | 56.1 |
| **Ethnicity, (%)** |  |  |
| Non-white | 2.7 | 4.8† |
| **Marital status, (%)** |  |  |
| Without conjugal life | 33.0 | 39.7† |
| **Schooling, (%)** |  |  |
| > 13 years | 33.1 | 27.8† |
| 12-13 years | 28.9 | 24.1† |
| ≤ 11 years | 38.0 | 48.1† |
| **Wealth, (%)** |  |  |
| Highest quintile | 22.9 | 18.2† |
| 2^nd^ quintile | 22.1 | 18.1† |
| 3^rd^ quintile | 20.8 | 19.8 |
| 4^th^ quintile | 18.3 | 19.4 |
| Lowest quintile | 13.9 | 20.6† |
| Not applicable | 2.0 | 3.9† |
| **Smoking, (%)** |  |  |
| Non-smoker | 38.5 | 35.7 |
| Ex-smoker | 50.2 | 51.0 |
| Smoker | 11.3 | 13.3† |
| **Alcohol intake, (%)** |  |  |
| Rarely/never | 18.7 | 20.7 |
| Frequently | 39.9 | 29.1† |
| Daily | 33.5 | 24.0† |
| Not applicable | 7.9 | 26.2† |
| **Physical activity, (%)** |  |  |
| High | 33.3 | 21.5† |
| Moderate | 47.9 | 41.9† |
| Low | 13.4 | 19.7† |
| Inactive | 5.4 | 16.9† |

**Note:** Quantitative variables expressed as mean ± standard deviation (SD). Qualitative variables expressed as percentages (%). † Statistically significant difference compared to included group (p < 0.05).

**Supplementary Table 2.** Clinical conditions, biochemical markers, anthropometric measurements, and performance measures of participants included versus excluded at baseline, ELSA Study (2012/13).

|  | **Included**  **(n = 5,520)** | **Excluded**  **(n = 3,649)** |
| --- | --- | --- |
| **Clinical conditions, (%)** |  |  |
| Hypertension | 37.4 | 45.0† |
| Diabetes mellitus | 9.5 | 15.6† |
| Cancer | 5.0 | 7.6† |
| Heart disease | 15.9 | 25.1† |
| Lung disease | 13.9 | 15.5 |
| Stroke | 3.4 | 7.7† |
| Osteoporosis | 8.0 | 9.9† |
| Osteoarthritis | 38.9 | 40.3 |
| Depressive symptoms | 11.3 | 15.1† |
| **Biochemical markers, (%)** |  |  |
| Total cholesterol, (mg/dL) | 214.1 ± 45.0 | 212.0 ± 53.0 |
| HDL, (mg/dL) | 64.3 ± 18.5 | 58.4 ± 20.3† |
| LDL, (mg/dL) | 124.4 ± 40.0 | 115.6 ± 43.0† |
| Triglycerides | 128.8 ± 63.0 | 203.8 ± 177.9† |
| **Anthropometrics and** **performance measures** |  |  |
| Body mass index, (kg/m²) | 28.0 ± 5.0 | 29.1 ± 5.8† |
| Normal weight | 27.5 | 23.8† |
| Underweight | 0.9 | 1.1 |
| Overweight | 42.2 | 37.5† |
| Obesity | 29.4 | 37.6† |
| Grip strength, (kg) | 30.8 ± 11.6 | 27.5 ± 12.5† |
| Memory performance, points | 11.1 ± 3.4 | 9.8 ± 4.1† |
| **Season of blood collection, (%)** |  |  |
| Spring | 7.5 | 6.2 |
| Summer | 23.8 | 22.8 |
| Autumn | 42.3 | 43.3 |
| Winter | 26.4 | 27.7 |
| **Vitamin D supplementation, (%)** | 4.4 | 4.6 |

**Note:** Quantitative variables are expressed as mean ± standard deviation (SD). Qualitative variables are expressed as percentages (%). HDL: high-density lipoprotein; LDL: low-density lipoprotein. NAO/VDS: Non-abdominal obesity/vitamin D sufficiency; NAO/VDI: Non-abdominal obesity/vitamin D insufficiency; NAO/VDD: Non-abdominal obesity/vitamin D deficiency; AO/VDS: Abdominal obesity/vitamin D sufficiency; AO/VDI: Abdominal obesity/vitamin D insufficiency; AO/VDD: Abdominal obesity/vitamin D deficiency. † Statistically significant difference compared with the included group (p < 0.05).
